# Psychopharmacological enhancement: a conceptual framework

**DOI:** 10.1186/1747-5341-7-5

**Published:** 2012-01-13

**Authors:** Dan J Stein

**Affiliations:** 1 Department of Psychiatry, University of Cape Town and Stellenbosch University, Groote Schuur Hospital, Anzio St, Cape Town 7925, South Africa

## Abstract

The availability of a range of new psychotropic agents raises the possibility that these will be used for enhancement purposes (smart pills, happy pills, and pep pills). The enhancement debate soon raises questions in philosophy of medicine and psychiatry (eg, what is a disorder?), and this debate in turn raises fundament questions in philosophy of language, science, and ethics. In this paper, a naturalistic conceptual framework is proposed for addressing these issues. This framework begins by contrasting classical and critical concepts of categories, and then puts forward an integrative position that is based on cognitive-affective research. This position can in turn be used to consider the debate between pharmacological Calvinism (which may adopt a moral metaphor of disorder) and psychotropic utopianism (which may emphasize a medical metaphor of disorder). I argue that psychiatric treatment of serious psychiatric disorders is justified, and that psychotropics are an acceptable kind of intervention. The use of psychotropics for sub-threshold phenomena requires a judicious weighing of the relevant facts (which are often sparse) and values.

## Introduction

Although psychotropics have been used for medicinal, recreational, and spiritual purposes since the dawn of humanity, the science of psychopharmacology is a relatively new one. In recent decades, however, there have been significant advances in understanding brain-mind structures and mechanisms, and a range of novel psychotropics have come to market. The availability of these agents raises the possibility that they will be used for enhancement purposes; that "smart pills" will be used to promote learning and clarify thinking, "happy pills" to increase mood and improve temperament, and "pep pills" to increase energy and maximize motivation.

The debate on so-called cosmetic psychopharmacology pits pharmacological Calvinism (which emphasizes the negative aspects of having a pill for every ill) against psychotropic utopianism (which underscore the positive aspects of enhancements). This debate immediately raises key questions in philosophy of medicine and psychiatry - in particular, what is a medical disorder, and what is a psychiatric disorder? These questions in turn raise fundament issues in philosophy of language, science, and ethics. In this paper, a naturalistic conceptual framework is proposed for addressing these issues and cosmetic psychopharmacology. This framework begins by contrasting classical and critical concepts of categories, and then puts forward an integrative position that is based on cognitive-affective research.

Before outlining this approach, however, let us consider 3 patient vignettes:

• Adam (aged 20) has severe social anxiety disorder with comorbid depression. As a child he had selective mutism, and had to repeat a year of schooling. Major depression began in adolescence. Social anxiety has meant that he was unable to go to college, or seek work. He has been in several psychotherapies, without clear effect. Treatment with an antidepressant has not yet been attempted.

• Beth (aged 24) has felt uncomfortable in social situations since adolescence. She worked as a secretary in a small town, and did fine. However, when her family moved to the city, and worked in a larger company, she was required to take part in small group activities. This led to significant distress, and she often thought of stopping work. She has never heard of social phobia, nor sought treatment.

• Cliff (aged 28) characterizes himself as shy. He works in a marketing company, and feels that his presentations are not nearly as good as they could be. He gets anxious, and stumbles more than others. It is important for him to come across more smoothly. He read about the efficacy of paroxetine for social anxiety disorder, and presented for treatment, wanting to try this medication.

## Classical vs critical approaches to categorization

An immediate question raised by these vignettes is the central question of philosophy of medicine and psychiatry, "what is a disorder?" A classical and a critical response to this question can be differentiated. Neither of these positions aligns with the work of any particular author, and each involves oversimplification of a vast literature; nevertheless, the contrast may have some heuristic value for developing an integrative conceptual framework [1].

A classical position argues that it is possible to develop necessary and sufficient criteria for defining not only disorder, but any particular category of psychopathology, such as depression (Adam), social phobia (Beth), or shyness (Cliff). After all, the concept of a square can be defined using necessary and sufficient criteria. Similarly, a disorder can be defined in essentialist terms, for example, as a malfunction. Such a position has roots in Plato, and can be traced through the work of the early Wittgenstein and logical positivism. A classical approach to categories holds that meaning can be fully specified in terms of our direct knowledge of the world, and subsequent definitions and logic.

A critical position on the other hand, argues that the definition of psychiatric disorders varies from place to place, and time to time. Similarly, when we consider the meaning of a construct such as a weed, the key considerations are the social practices of gardeners, and these in turn reflect a particular context. Thus, our concepts of disorder are best understood as social constructions. Such a position has roots in the work of writers like Vico and Herder, and can be traced through the work of the later Wittgenstein, and much continental philosophy. A critical approach argues that language reflects a speakers' way of life, and meaning cannot be reduced to formal rules.

An integrative position emphasizes that a range of cognitive-affective data can be used to understand the category of disorders. Although categories reflect social activities, humans are able to investigate their underlying structures and mechanisms [2], and so can reasonably debate classifications. Birds, for example, fall into those that are viewed as typical (eg robins), that those are less typical (eg owls) and those that are atypical (eg ostriches). Nevertheless, various data indicate that ostriches can validly be classified as birds. Such a position again has early roots, and has been articulated by various modern philosophers [3]. An integrative approach argues that categories are embodied in our brain-minds, and in our social activities, but that they can be reasonably debated.

Although it might be useful to use empirical data to assess which disorders are seen as typical, less typical, and typical, examples of these come readily to mind. In typical disorders (eg infection, posttraumatic stress disorder), metaphors of warfare/contamination, of impediment/breakdown, or of imbalance are used. In these MEDICAL metaphors, disorder is caused by an external agent, which treatment then addresses. In atypical disorders (eg obesity, impulsive-aggression), these metaphors fit less well (eg the role of external agents is less clear, or treatment encourages personal responsibility). Thus a MORAL metaphor may also be employed.

## Implications: Entities, accounts, values

The contrast drawn here between classical and critical concepts of disorders leads in turn to a range of fundamental questions about the entities in the world, about accounts of these entities, and about the way in which we value them. A brief paper cannot due full justice to the broad range of metaphysical, epistemological, and ethical literature that is relevant to these questions. Nevertheless, there may be some heuristic value in contrasting certain key classical versus critical positions, and putting forward an integrative position based on cognitive-affective data [1].

### a) Entities

A position which conceptualizes categories in essentialist terms leads in turn to a classical approach to a range of other entities that are relevant to debates about cosmetic psychopharmacology. These include personal identity, emotion, and psychotropics. A classical view may attempt to define the self in an essentialist way (consider Descartes' view of the soul), to contrast emotion clearly with reason (a cognitivist view regards emotions in terms of beliefs and desires), and to delineate rigorously medications from enhancements (the former are used to treat medical disorders, the latter are used for other purposes).

In contrast, a view which regards categories as social constructions leads in turn to a critical approach to this range of entities. A critical view may argue that the self is not fixed and immutable (consider views of the self as narrative), may emphasize the non-essentialist nature of emotion (emphasizing, for example, feelings as socially constructed), and may conclude that there are no rigorous boundaries between psychiatric drugs, illicit substances, and nutraceuticals (the distinctions are porous, and vary from time to time and place to place). (There is a tendency, though, for critical authors to regard psychiatric drugs as non-progressive technologies of the self, while viewing illicit substances as promising psychedelics and entheogens).

A perspective which regards categories as social constructed, but also as potentially informed by knowledge of their underlying structures and mechanisms, leads in turn to an integrative approach to the self, to emotion, and to medication. While the self is certainly auto-constructed, it is possible to make judgments about the authenticity of particular selves (for example, a patient with depression may regard their current self as inauthentic). Emotion and reason are intertwined in complex ways (and it is possible to determine in a reasonable way whether social anxiety is appropriate or not). While social convention draws particular boundaries between different kinds of psychotropic, it turns out that specific brain-mind structures and mechanisms determine whether or not such molecules are effective, lead to dependence, or have other adverse events.

### b) Accounts

A classical position can also be formulated for addressing explanations of the world in general, and of the brain-mind in particular. Positivist philosophers, for example, have emphasized the uniformity of the laws of nature, arguing that the scientific method involves inducing laws from empirical data. Similarly, behaviourism, symbolic cognitivism, and certain approaches within psychoanalysis have emphasized the importance of attempting to find regular laws which cover brain-mind phenomena and psychiatric treatments. A classical approach within psychopharmacology views the placebo effect as "noise" which must subtracted out in order to see the real "signals" in the data. Finally, a classical approach to evolutionary psychiatry emphasizes evolved mental modules, and their dysfunction in disorder.

In contrast, a critical position argues that when it comes to human phenomena, including brain-mind phenomena, our approach must focus on understanding (verstehen) rather than explanation (erklären). A focus on narratives and their interpretation is key in the human sciences. Similarly, critics of behaviourism, situated cognitivism, and hermeneutic approaches within psychoanalysis have emphasized the importance of human understanding and of social context in approaching both human psychopathology and clinical intervention. A critical approach may emphasize the strength of the placebo effect, and the way in which it points to the importance of meaning in the doctor-patient relationship. A critical approach may argue that humans have evolved to use language, and that what counts as human nature varies from time to time and place to place.

A perspective which emphasizes both verstehen and erklären in approaching complex brain-mind phenomena arguably leads to more integrative accounts. Psychiatry has developed increasingly sophisticated accounts of psychopathology and of treatment, incorporating both top-down perspectives (emphasizing social context) and bottom-up accounts (emphasizing genes and proteins). Similarly, a complex account of placebo and nocebo responses is now possible; this includes awareness not only of the importance of the doctor-patient relationship, but also the way in which this is mediated by specific neurocircuitry. Evolutionary theory both contributes to the richness of our scientific accounts, including our account of human nature, and simultaneously clarifies that humans have socially constructed natures.

### c) Values

A classical approach would take the view that decisions about ethics in general and bioethics in particular can be made on the basis of moral laws. Thinkers from Plato through Kant have described the rationality of such universal and fixed rules, with more recent philosophers such as Rawls viewing moral theory as a kind of grammar [4]. On the basis of a determination about whether a relevant condition involves say a dysfunction (a matter of fact), and a determination about whether say maximum benefit for the community is being achieved (a matter of value), for example, a principled decision can be made as to whether or not to treat. In this classic view, modern bioethics is able to resolve questions raised by the practice of medicine, including problems raised by new technologies, by means of moral rules, which apply universally, and sustain the important distinction between facts and values.

In contrast, a critical position emphasizes that such a view is unrealizable given that decisions about ethics and bioethics reflect the concerns and values of the particular historical and geographical contexts in which they are made. Hegel, for example, argued that a systems of laws loses its meaning when attempts are made to abstract it from its grounding in the ethical life of a particular historical community. Emotional reactions are normal and valuable phenomena, our constructs (or "idioms") of distress are culture-bound, and such distress need not necessarily be medicalized. Decisions about ethics and bioethics reflect the concerns and values of the particular historical and geographical contexts in which they are made. A close examination of the words and metaphors used in moral reasoning and of the nature of bioethics refutes the notion of a fact-value distinction.

An integrative position argues that insofar as decisions about ethics and bioethics are informed by universal biologically-based considerations there exists the potential for minimal standards of agreement about ethics and about values, and it is possible to debate reasonably in order to ensure that good bioethics decisions are reached. A naturalist ethics can be traced to Aristotle, who as a biologist attempted to give an account of the good that was both local and particular, and yet also cosmic and universal [5]. For example, there is near universal agreement that when physicians relieve pain this is a good thing, and yet it is also possible to argue reasonably for the importance of not relieving pain or suffering in particular contexts. Naturalist positions range from a broad approach (which holds that a naturalist approach to philosophical inquiry in general is adequate for moral philosophy) to a narrower view (which holds that there are natural moral facts and properties). Here it is necessary only to highlight the complexity of facts about the human species and the way these interact with human values, noting that this is consistent with a non-reductionistic naturalism.

## Three vignettes

With this conceptual framework in mind, let us return to our 3 vignettes.

### a) Adam

From a classical perspective, Adam clearly suffers from a well-described medical disorder (depression). We can readily make objective assessments of his abnormal sense of self, his low mood, and of the effects of particular antidepressant medications. Fortunately psychiatry has been able to demonstrate that depression is associated with biochemical disturbances, and that antidepressant agents are able to improve symptoms. The standard moral rules of good medicine apply also in psychiatry, and there is a clear-cut case for treating Adam's medical disorder with modern psychotropic agents as soon as possible.

From a critical perspective, there are many other ways of construing Adam. A diagnosis of depression tells us much about the particular social context of Western medicine, but relatively little about the nature of the distress which he is suffering. To understand fully his self, his mood, and his response to intervention, we need to address the nature of his circumstances and the meaning of his response. Indeed, this is the role of the clinician; to assist Adam to understand his context, and to help him construct a response to it. Medicalization of this process is not necessarily useful, and whatever approach we take in helping Adam reflects the particularity of our own value system.

From an integrative perspective, we can debate rationally whether or not Adam has a disorder, and similarly assess the extent to which his self-alteration, mood, and response to medication are consistent with a MEDICAL metaphor. Certainly, depression is associated with significant disability and suffering, and current interventions are effective and cost-efficient. Furthermore, we increasingly understand the specific structures and mechanisms which underlie depression, and the way in which interventions act to normalize such structures and mechanisms. While moral decision-making cannot be reduced to algorithms, neither is it entirely relativistic; a reasonable decision to treat Adam emerges from a consideration of the relevant data and values.

### b) Beth

When it comes to Beth, many have argued that social anxiety disorder (or social phobia), like depression, is a medical disorder that deserves clinical intervention. From a classical perspective, social anxiety disorder like depression can be thought of in terms of a "chemical imbalance". At the same time, critics have argued that the concept of social anxiety disorder represents "disease mongering", that there is no evidence that social anxiety disorder involves real dysfunction, and that we need to be sceptical about the need for medical intervention. Certainly, social anxiety disorder appears somewhat different from acute depression, in that it is often a lifelong condition that alters the very nature of the self. My own view is that there now a considerable set of data which highlights the suffering and disability of those with social anxiety disorder, points to underlying psychobiological alterations, and emphasizes the efficacy and cost-efficiency of treatment. Thus an integrated approach supports the validity of using a MEDICAL metaphor for social anxiety disorder. Such a view is equally accepting of both pharmacotherapy and psychotherapy.

### c) Cliff

A classical view of medicine has tended to focus on disease rather than on physical or mental well-being. One reason for this may be the difficulty in finding consensus on what might constitute a decrease in well-being and in reliably measuring subtle forms of decreased well-being. There are, however, also ethical arguments for making a distinction between doctoring (treating dysfunction) and schmoctering (focusing on enhancement) and avoiding the latter [6]. Some authors [7,8] argue on the basis of the principles of distributive justice that society need only provide resources for the former. Others (bioconservatives) argue that when medicine focuses on enhancement, it risks upsetting the natural order of things. In the United States, the President's Council on Bioethics has argued that "[T]he naturalness of means matters. It lies not in the fact that the assisting drugs or devices are artefacts, but in the danger of violating or deforming the nature of human agency ... biotechnology interventions act directly on the human body and mind to bring about their effects on a passive subject [9].

From a critical perspective, enhancement technologies, ranging from cosmetic surgery to cosmetic psychopharmacology, reinforce particular social constructions and values. The extension of medicine to include enhancement technologies is consistent with its role in decreasing social deviance, and is problematic insofar as it focuses on pathology and ignores resilience, overemphasizes technology and dehumanizes people, and ignores our overall physical, psychological, and spiritual well-being. Some psychotherapies may be useful in fostering self-knowledge and even self-transcendence [10], and some psychotropics (eg substances) may be of interest insofar as they allow an interrogation and deconstruction of "reality". However, just as cosmetic surgery reinforces particular social values (for example, equating women's looks with their value), so cosmetic psychopharmacology acts primarily as a conservative force, promoting inauthenticity and interfering with self-understanding. As Murray [11], for example, writes "Medicine is now the problem of the self; and medicine, we are told is the necessary solution to the problem - a problem that this medical discourse has in fact secretly produced and systematically obscured ...the modern self remains constrained by a medical morality: I am morally remiss, my life is a life unworthy of living if I fail to submit to medical examinations, to doctors' and psychiatrists' recommendations".

Williams [12] compares hyper-traditional societies, where there has been no exposure to contrasting views, with our modern world, where there is increasing exposure to a range of opinions. In a hyper-traditional society, there may be little reflexive thought on where the boundaries of treatment should lie, the natural order of things is obvious. But since the dawn of the modern world, the lines between doctoring and schmoctoring have been open to debate, and ever shifting. An integrative perspective would be wary of any attempt to find a universal rule that differentiated between treatment and enhancement, or conversely, that rejected any possibility of a reasonable decision about where this line should be drawn. Again, the argument can be made that for any particular individual, conceptual and empirical work can help determine what the best intervention is, at any particular point in time. Still, although advances in biotechnology may be useful for a specific individual, the practices that they bring into play may also come with important costs.

There is a tension in Western society between values of self-improvement and self-creation, which would encourage people to use novel technologies including medical enhancement, and values around tradition and nature and about true self-discovery which argue that such changes are not authentic [6,13]. As Parens writes, "As one side emphasises our obligation to remember that life is a gift and that we need to learn to let things be, the other emphasizes our obligation to transform that gift and to exhibit our creativity" [14]. In Huxley's "Island" the moksha-medicine suggests the possibility of using medication to enhance authenticity, but in his "Brave New World" soma serves to illustrate how psychopharmacological enhancement can be dehumanizing [15]. Given the emphasis that we place on effort in assigning responsibility for achievement, many view enhancement as representing diminished agency on the part of the person whose achievement is enhanced. Sandel, a member of the President's Council, counter-argues however that both enhancement technologies, and also particularly high pressure kinds of parenting (or "hyperparenting"), represent not so much a loss of agency, but rather hyperagency - an aspiration to remake nature, including human nature [16]. Mastery over nature, and self-improvement, have obvious benefits. But there is also the risk that viewing our talents solely as achievements for which we are responsible, leads to hubris about ourselves (we are self-made and self-sufficient rather than merely fortunate), and a lack of solidarity with others less fortunate (who are deserving of their fate rather than merely unfortunate) [16].

Johnson has argued persuasively that moral reasoning is a special case of general (cognitive-affective) reasoning, and therefore must rely not so much on algorithmic rules, as on the use of embodied metaphors [17]. It is precisely when our concepts of what is natural are challenged, for example, by new technologies, that the question of which metaphor to apply becomes particularly crucial. Thus, in the case of cosmetic psychopharmacology (and other new technologies), we are left with the question of whether the MEDICAL metaphor of self-treatment (typically applied when some kind of unnatural breakdown occurs) or the MORAL metaphor of self-discovery (typically applied to everyday or apparently natural life), or a combination of the two, is more appropriate. Life often presents us with conflicting moral solutions that cannot be hierarchically ordered by any universal principle. Those using psychotropics may find themselves using different metaphors at different times. Indeed, in medical practice in general, and in cosmetic psychopharmacology in particular, the right balance between over-use and under-use of medical metaphors, remains a matter of judgment.

Routine vaccination and fluoride supplementation are paradigmatic of health care interventions that are undertaken in healthy children to strengthen them (rather than to treat a disorder), and that are now correctly viewed as good doctoring. Similarly, folic acid is added to food to prevent neural tube defects in newborns, and vitamin supplementation may be recommended in some healthy adults. Although the benefits of such practices have on balance been enormous, there have been significant costs for particular individuals (for example, those who have serious adverse reactions to vaccination). As interventions for high cholesterol and high blood pressure have been introduced, and become cheaper, so our cut-points for deciding who to treat have become less and less conservative. In the past it has been argued that antidepressants are only useful in those with major depression, but there is now evidence that minor disorders are accompanied by significant disability and also respond to these agents. The risk-benefit of such interventions can be calculated using statistical measures such as numbers needed to treat and numbers needed to harm.

Although the analysis here has focus on disorder, analogous considerations would argue that no essentialist definition of health is possible, and its boundaries must be decided as reasonably as possible. The construct of "well-being" is often a component of our cognitive-affective maps for decision-making and moral judgments [3], and may be more relevant to considering "enhancements" rather than "treatments". Given that humans do have some design flaws, and given advances in pharmaceuticals and psychotropics, it is not unlikely that more and more of these agents will be appropriately prescribed to more and more people for the purposes of well-being. It is not at all clear that there is any a priori ethical reason for rejecting a shift from a hyper-traditional view of doctoring, and moving to one that allows the employment of biotechnological advances to improve well-being [18]. Arguments that such advances threaten the natural order, for example, may simply reflect culture-specific views about what sort of conditions are needed for a good life [19].

On the other hand, there may be important costs associated with this kind of extension of doctoring. Given how well humans have been designed by evolution, the difficulties facing innovative science, and the high placebo response in those who suffer only mild impairment, it is hard to generate data showing that interventions to improve physical or psychological well-being are in fact effective and cost-effective. A growing empirical literature on happiness and well-being [20] supports the view that gaining achievement at the cost of social connectedness is ultimately experienced as disadvantageous. Furthermore, raising expectations about the existence and value of enhancement technologies may be one of the reasons for the so-called "paradox of health" in contemporary societies; where populations that are in fact well, but also well-informed about pathology, complain more about disorder than unwell but uninformed groups [21]. Thus, the employment of biotechnological advances to improve well-being may at times be bad doctoring.

Neither evolutionary theory, nor blanket ethical principles, provide sufficient guidance for deciding on whether or not to medically intervene. There is general agreement that prescribing psychotropics for depression is useful, that providing psychotropics to an abused woman in order to take away the pain in her current abusive relationship without supporting attempts to change or discontinue the relationship is bad doctoring, and that using steroids with terrible side effects to enhance athletic performance is bad schmoctoring. On the other hand, in some cases it would seem good judgment to try an "enhancement" in the hope that it would in fact serve as a useful "treatment". This position is consistent with a realist view that emphasizes an individualist approach to clinical decision-making, and that is neither overly optimistic nor unduly pessimistic about biotechnological advances.

Just as the media cannot be held wholly responsible for modern ideals of beauty, so the pharmaceutical industry cannot be said to have entirely manufactured our concepts of either psychiatric disorder or mental fitness. On the other hand, just as it is important to interrogate media constructs of beauty and to offer alternatives, so it is crucial to assess the extent to which the claims of the pharmaceutical, alternative, and indigenous medicines industries and other promoters of psychotropics go beyond the relevant science, and to offer a range of alternatives to those who may understandably want to use these agents to change their lives for the better. Psychopharmacology has the potential to liberate us by reducing the limits imposed on us by disorder, but to imprison us by imposing societal demands [22]. In weighing up alternatives, it is certainly important to remember the excesses of past marketing campaigns, and the overoptimism of researchers and clinicians about novel breakthroughs in psychopharmacology [23]. At the same time, and for particular individuals, at particular times, there is no reason to remain completely closed to the possibility that a specific psychotropic will turn out to be remarkably helpful. Good clinical practice invariably involves the kind of equipoise that comes from successfully balancing the perennial tensions described in this paper between the objective and subjective, explanation and understanding, and conflicting values.

We now know that specific genetic variants, in interaction with particular environments, determine set-points in various neuronal circuits. We can no longer infer that all people experience pain or anxiety in the same way. Similarly, for particular individuals, certain psychotropics may be useful; people with the val-val polymorphism of the catechol-0-methyltransferase (COMT) gene are characterized by lower prefrontal dopamine levels and worse performance on working memory tasks, and may do well on certain medications - whereas people with the met-met COMT polymorphism have higher prefrontal dopamine levels and better performance, and may respond less well to the same agents [24]. On the basis of existing cognitive-affective science it is possible to predict that in the future, physicians and psychopharmacologists will obtain detailed profiling of patients' genetic variants, and offer an individualized battery of medications and psychotropics. There is the possibility that this will be done not merely to reduce conditions such as social anxiety and impulsivity, but also to increase well-being and enhance cognition.

On the other hand, it is important to emphasize the complexity of our genes and their interactions, the unintended consequences of medication use, and the difficulties of coming up with elixirs that can outdo our evolutionary design. An intervention that acts on only a few receptor targets may ultimately affect multiple processes in unpredictable ways. Just like the earlier "clinical gaze" [25], so the more recent "molecular gaze" [11,26] has its limitations. Indeed, despite much back-slapping about the value of current psychopharmacology, there has also been a great deal of exaggeration [23]. It is notable that currently available medications all work via only a few mechanisms, reflecting how difficult it is to make real breakthroughs in our pharmacopoeia. Similarly, current medications act on a fairly narrow range of phenomena (primarily mood and anxiety), and are relatively unhelpful for changing a range of other traits that may play a role in determining well-being (eg empathy). Again, to date, it is not yet clear that available agents are better than, say, caffeine, or exercise, as cognitive enhancers. Thus, despite the already widespread use of psychotropics to improve well-being and enhance cognition, only time will tell how valuable for humans the field of cosmetic psychopharmacology will ultimately be.

The selective serotonin reuptake inhibitors (SSRIs) deserve particular mention, as they have been key to this debate since the work of Kramer [27]. It should be emphasized that to date there is relatively little empirical data on the use of psychotropics such as SSRIs in healthy volunteers. It is interesting that some controlled research demonstrates subtle changes in emotional processing and social behaviour in normals, sometimes outside of the awareness of subjects. Nevertheless, closer examination of such phenomena, as well as of the use of SSRIs in those with more significant symptomatology (even if it did not meet criteria for "major depression" or similar disorders) would be necessary to determine whether it was worthwhile or not. Currently there is insufficient evidence to argue that administration of an SSRI to healthy subjects results in increased well-being [28]. Conversely, there is some evidence that SSRIs can flip predisposed people into a hypomanic state. In the absence of knowledge of pharmacogenetic predictors of response, and likely even if these were available, the ultimate value of a particular psychotropic for a patient may be difficult to predict until after it has been prescribed. Nutraceuticals, such as caffeine, and psychostimulant medications are widely used to enhance attention and memory, but again the evidence-base to support the value of newer psychotropics as cognitive enhancers is currently sparse [29].

In summary, a classical approach to ethics in general, and to bioethics in particular, attempts to find moral rules for particular situations. In contrast, a critical approach argues that values inhere in our ways of life, and that ethics can't be algorithmized. An integrative approach would argue that yes, values inhere in our way of life but first, these are closely related to our human nature - many would agree that pain is bad - and second they can be reasonably (ie cognitive-affectively) debated against the backdrop of particular circumstances (there are particular contexts in which pain may in fact be a reasonable choice). We cannot always reach universal conclusions about the nature of humans in general, or even the value of psychotropics (good or bad) in a specific patient. In the more subtle cases of cosmetic psychopharmacology a lack of ethical convergence is reinforced by a relative absence of empirical data. Nevertheless, in particular instances we can reach reasonable decisions - some lives are better than others. Relevant considerations for moral reasoning-imagining about enhancements include both the potential value of stoic acceptance of life's fate (including imperfections and aging, day-to-day distress, and idiosyncracies in personality), as well as the potential value of attempts to change the hand one is dealt (whether by virtue of pharmacotherapy, psychotherapy, or non-medical means). Decisions about whether or not to use psychotropics are likely to remain controversial and given the relative complexity and difficulty of such decisions, this is appropriate.

## Three vignettes

With this conceptual framework in mind, let us return to our 3 vignettes.

### a) Adam

From a classical perspective, Adam clearly suffers from a well-described medical disorder (depression). We can readily make objective assessments of his abnormal sense of self, his low mood, and of the effects of particular antidepressant medications. Fortunately psychiatry has been able to demonstrate that depression is associated with biochemical disturbances, and that antidepressant agents are able to improve symptoms. The standard moral rules of good medicine apply also in psychiatry, and there is a clear-cut case for treating Adam's medical disorder with modern psychotropic agents as soon as possible.

From a critical perspective, there are many other ways of construing Adam. A diagnosis of depression tells us much about the particular social context of Western medicine, but relatively little about the nature of the distress which he is suffering. To understand fully his self, his mood, and his response to intervention, we need to address the nature of his circumstances and the meaning of his response. Indeed, this is the role of the clinician; to assist Adam to understand his context, and to help him construct a response to it. Medicalization of this process is not necessarily useful, and whatever approach we take in helping Adam reflects the particularity of our own value system.

From an integrative perspective, we can debate rationally whether or not Adam has a disorder, and similarly assess the extent to which his self-alteration, mood, and response to medication are consistent with a MEDICAL metaphor. Certainly, depression is associated with significant disability and suffering, and current interventions are effective and cost-efficient. Furthermore, we increasingly understand the specific structures and mechanisms which underlie depression, and the way in which interventions act to normalize such structures and mechanisms. While moral decision-making cannot be reduced to algorithms, neither is it entirely relativistic; a reasonable decision to treat Adam emerges from a consideration of the relevant data and values.

### b) Beth

When it comes to Beth, many have argued that social anxiety disorder (or social phobia), like depression, is a medical disorder that deserves clinical intervention. From a classical perspective, social anxiety disorder like depression can be thought of in terms of a "chemical imbalance". At the same time, critics have argued that the concept of social anxiety disorder represents "disease mongering", that there is no evidence that social anxiety disorder involves real dysfunction, and that we need to be sceptical about the need for medical intervention. Certainly, social anxiety disorder appears somewhat different from acute depression, in that it is often a lifelong condition that alters the very nature of the self. My own view is that there now a considerable set of data which highlights the suffering and disability of those with social anxiety disorder, points to underlying psychobiological alterations, and emphasizes the efficacy and cost-efficiency of treatment. Thus an integrated approach supports the validity of using a MEDICAL metaphor for social anxiety disorder. Such a view is equally accepting of both pharmacotherapy and psychotherapy.

### c) Cliff

A classical view of medicine has tended to focus on disease rather than on physical or mental well-being. One reason for this may be the difficulty in finding consensus on what might constitute a decrease in well-being and in reliably measuring subtle forms of decreased well-being. There are, however, also ethical arguments for making a distinction between doctoring (treating dysfunction) and schmoctering (focusing on enhancement) and avoiding the latter [6]. Some authors [7,8] argue on the basis of the principles of distributive justice that society need only provide resources for the former. Others (bioconservatives) argue that when medicine focuses on enhancement, it risks upsetting the natural order of things. In the United States, the President's Council on Bioethics has argued that "[T]he naturalness of means matters. It lies not in the fact that the assisting drugs or devices are artefacts, but in the danger of violating or deforming the nature of human agency ... biotechnology interventions act directly on the human body and mind to bring about their effects on a passive subject [9].

From a critical perspective, enhancement technologies, ranging from cosmetic surgery to cosmetic psychopharmacology, reinforce particular social constructions and values. The extension of medicine to include enhancement technologies is consistent with its role in decreasing social deviance, and is problematic insofar as it focuses on pathology and ignores resilience, overemphasizes technology and dehumanizes people, and ignores our overall physical, psychological, and spiritual well-being. Some psychotherapies may be useful in fostering self-knowledge and even self-transcendence [10], and some psychotropics (eg substances) may be of interest insofar as they allow an interrogation and deconstruction of "reality". However, just as cosmetic surgery reinforces particular social values (for example, equating women's looks with their value), so cosmetic psychopharmacology acts primarily as a conservative force, promoting inauthenticity and interfering with self-understanding. As Murray [11], for example, writes "Medicine is now the problem of the self; and medicine, we are told is the necessary solution to the problem - a problem that this medical discourse has in fact secretly produced and systematically obscured ...the modern self remains constrained by a medical morality: I am morally remiss, my life is a life unworthy of living if I fail to submit to medical examinations, to doctors' and psychiatrists' recommendations".

Williams [12] compares hyper-traditional societies, where there has been no exposure to contrasting views, with our modern world, where there is increasing exposure to a range of opinions. In a hyper-traditional society, there may be little reflexive thought on where the boundaries of treatment should lie, the natural order of things is obvious. But since the dawn of the modern world, the lines between doctoring and schmoctoring have been open to debate, and ever shifting. An integrative perspective would be wary of any attempt to find a universal rule that differentiated between treatment and enhancement, or conversely, that rejected any possibility of a reasonable decision about where this line should be drawn. Again, the argument can be made that for any particular individual, conceptual and empirical work can help determine what the best intervention is, at any particular point in time. Still, although advances in biotechnology may be useful for a specific individual, the practices that they bring into play may also come with important costs.

There is a tension in Western society between values of self-improvement and self-creation, which would encourage people to use novel technologies including medical enhancement, and values around tradition and nature and about true self-discovery which argue that such changes are not authentic [6,13]. As Parens writes, "As one side emphasises our obligation to remember that life is a gift and that we need to learn to let things be, the other emphasizes our obligation to transform that gift and to exhibit our creativity" [14]. In Huxley's "Island" the moksha-medicine suggests the possibility of using medication to enhance authenticity, but in his "Brave New World" soma serves to illustrate how psychopharmacological enhancement can be dehumanizing [15]. Given the emphasis that we place on effort in assigning responsibility for achievement, many view enhancement as representing diminished agency on the part of the person whose achievement is enhanced. Sandel, a member of the President's Council, counter-argues however that both enhancement technologies, and also particularly high pressure kinds of parenting (or "hyperparenting"), represent not so much a loss of agency, but rather hyperagency - an aspiration to remake nature, including human nature [16]. Mastery over nature, and self-improvement, have obvious benefits. But there is also the risk that viewing our talents solely as achievements for which we are responsible, leads to hubris about ourselves (we are self-made and self-sufficient rather than merely fortunate), and a lack of solidarity with others less fortunate (who are deserving of their fate rather than merely unfortunate) [16].

Johnson has argued persuasively that moral reasoning is a special case of general (cognitive-affective) reasoning, and therefore must rely not so much on algorithmic rules, as on the use of embodied metaphors [17]. It is precisely when our concepts of what is natural are challenged, for example, by new technologies, that the question of which metaphor to apply becomes particularly crucial. Thus, in the case of cosmetic psychopharmacology (and other new technologies), we are left with the question of whether the MEDICAL metaphor of self-treatment (typically applied when some kind of unnatural breakdown occurs) or the MORAL metaphor of self-discovery (typically applied to everyday or apparently natural life), or a combination of the two, is more appropriate. Life often presents us with conflicting moral solutions that cannot be hierarchically ordered by any universal principle. Those using psychotropics may find themselves using different metaphors at different times. Indeed, in medical practice in general, and in cosmetic psychopharmacology in particular, the right balance between over-use and under-use of medical metaphors, remains a matter of judgment.

Routine vaccination and fluoride supplementation are paradigmatic of health care interventions that are undertaken in healthy children to strengthen them (rather than to treat a disorder), and that are now correctly viewed as good doctoring. Similarly, folic acid is added to food to prevent neural tube defects in newborns, and vitamin supplementation may be recommended in some healthy adults. Although the benefits of such practices have on balance been enormous, there have been significant costs for particular individuals (for example, those who have serious adverse reactions to vaccination). As interventions for high cholesterol and high blood pressure have been introduced, and become cheaper, so our cut-points for deciding who to treat have become less and less conservative. In the past it has been argued that antidepressants are only useful in those with major depression, but there is now evidence that minor disorders are accompanied by significant disability and also respond to these agents. The risk-benefit of such interventions can be calculated using statistical measures such as numbers needed to treat and numbers needed to harm.

Although the analysis here has focus on disorder, analogous considerations would argue that no essentialist definition of health is possible, and its boundaries must be decided as reasonably as possible. The construct of "well-being" is often a component of our cognitive-affective maps for decision-making and moral judgments [3], and may be more relevant to considering "enhancements" rather than "treatments". Given that humans do have some design flaws, and given advances in pharmaceuticals and psychotropics, it is not unlikely that more and more of these agents will be appropriately prescribed to more and more people for the purposes of well-being. It is not at all clear that there is any a priori ethical reason for rejecting a shift from a hyper-traditional view of doctoring, and moving to one that allows the employment of biotechnological advances to improve well-being [18]. Arguments that such advances threaten the natural order, for example, may simply reflect culture-specific views about what sort of conditions are needed for a good life [19].

On the other hand, there may be important costs associated with this kind of extension of doctoring. Given how well humans have been designed by evolution, the difficulties facing innovative science, and the high placebo response in those who suffer only mild impairment, it is hard to generate data showing that interventions to improve physical or psychological well-being are in fact effective and cost-effective. A growing empirical literature on happiness and well-being [20] supports the view that gaining achievement at the cost of social connectedness is ultimately experienced as disadvantageous. Furthermore, raising expectations about the existence and value of enhancement technologies may be one of the reasons for the so-called "paradox of health" in contemporary societies; where populations that are in fact well, but also well-informed about pathology, complain more about disorder than unwell but uninformed groups [21]. Thus, the employment of biotechnological advances to improve well-being may at times be bad doctoring.

Neither evolutionary theory, nor blanket ethical principles, provide sufficient guidance for deciding on whether or not to medically intervene. There is general agreement that prescribing psychotropics for depression is useful, that providing psychotropics to an abused woman in order to take away the pain in her current abusive relationship without supporting attempts to change or discontinue the relationship is bad doctoring, and that using steroids with terrible side effects to enhance athletic performance is bad schmoctoring. On the other hand, in some cases it would seem good judgment to try an "enhancement" in the hope that it would in fact serve as a useful "treatment". This position is consistent with a realist view that emphasizes an individualist approach to clinical decision-making, and that is neither overly optimistic nor unduly pessimistic about biotechnological advances.

Just as the media cannot be held wholly responsible for modern ideals of beauty, so the pharmaceutical industry cannot be said to have entirely manufactured our concepts of either psychiatric disorder or mental fitness. On the other hand, just as it is important to interrogate media constructs of beauty and to offer alternatives, so it is crucial to assess the extent to which the claims of the pharmaceutical, alternative, and indigenous medicines industries and other promoters of psychotropics go beyond the relevant science, and to offer a range of alternatives to those who may understandably want to use these agents to change their lives for the better. Psychopharmacology has the potential to liberate us by reducing the limits imposed on us by disorder, but to imprison us by imposing societal demands [22]. In weighing up alternatives, it is certainly important to remember the excesses of past marketing campaigns, and the overoptimism of researchers and clinicians about novel breakthroughs in psychopharmacology [23]. At the same time, and for particular individuals, at particular times, there is no reason to remain completely closed to the possibility that a specific psychotropic will turn out to be remarkably helpful. Good clinical practice invariably involves the kind of equipoise that comes from successfully balancing the perennial tensions described in this paper between the objective and subjective, explanation and understanding, and conflicting values.

We now know that specific genetic variants, in interaction with particular environments, determine set-points in various neuronal circuits. We can no longer infer that all people experience pain or anxiety in the same way. Similarly, for particular individuals, certain psychotropics may be useful; people with the val-val polymorphism of the catechol-0-methyltransferase (COMT) gene are characterized by lower prefrontal dopamine levels and worse performance on working memory tasks, and may do well on certain medications - whereas people with the met-met COMT polymorphism have higher prefrontal dopamine levels and better performance, and may respond less well to the same agents [24]. On the basis of existing cognitive-affective science it is possible to predict that in the future, physicians and psychopharmacologists will obtain detailed profiling of patients' genetic variants, and offer an individualized battery of medications and psychotropics. There is the possibility that this will be done not merely to reduce conditions such as social anxiety and impulsivity, but also to increase well-being and enhance cognition.

On the other hand, it is important to emphasize the complexity of our genes and their interactions, the unintended consequences of medication use, and the difficulties of coming up with elixirs that can outdo our evolutionary design. An intervention that acts on only a few receptor targets may ultimately affect multiple processes in unpredictable ways. Just like the earlier "clinical gaze" [25], so the more recent "molecular gaze" [11,26] has its limitations. Indeed, despite much back-slapping about the value of current psychopharmacology, there has also been a great deal of exaggeration [23]. It is notable that currently available medications all work via only a few mechanisms, reflecting how difficult it is to make real breakthroughs in our pharmacopoeia. Similarly, current medications act on a fairly narrow range of phenomena (primarily mood and anxiety), and are relatively unhelpful for changing a range of other traits that may play a role in determining well-being (eg empathy). Again, to date, it is not yet clear that available agents are better than, say, caffeine, or exercise, as cognitive enhancers. Thus, despite the already widespread use of psychotropics to improve well-being and enhance cognition, only time will tell how valuable for humans the field of cosmetic psychopharmacology will ultimately be.

The selective serotonin reuptake inhibitors (SSRIs) deserve particular mention, as they have been key to this debate since the work of Kramer [27]. It should be emphasized that to date there is relatively little empirical data on the use of psychotropics such as SSRIs in healthy volunteers. It is interesting that some controlled research demonstrates subtle changes in emotional processing and social behaviour in normals, sometimes outside of the awareness of subjects. Nevertheless, closer examination of such phenomena, as well as of the use of SSRIs in those with more significant symptomatology (even if it did not meet criteria for "major depression" or similar disorders) would be necessary to determine whether it was worthwhile or not. Currently there is insufficient evidence to argue that administration of an SSRI to healthy subjects results in increased well-being [28]. Conversely, there is some evidence that SSRIs can flip predisposed people into a hypomanic state. In the absence of knowledge of pharmacogenetic predictors of response, and likely even if these were available, the ultimate value of a particular psychotropic for a patient may be difficult to predict until after it has been prescribed. Nutraceuticals, such as caffeine, and psychostimulant medications are widely used to enhance attention and memory, but again the evidence-base to support the value of newer psychotropics as cognitive enhancers is currently sparse [29].

In summary, a classical approach to ethics in general, and to bioethics in particular, attempts to find moral rules for particular situations. In contrast, a critical approach argues that values inhere in our ways of life, and that ethics can't be algorithmized. An integrative approach would argue that yes, values inhere in our way of life but first, these are closely related to our human nature - many would agree that pain is bad - and second they can be reasonably (ie cognitive-affectively) debated against the backdrop of particular circumstances (there are particular contexts in which pain may in fact be a reasonable choice). We cannot always reach universal conclusions about the nature of humans in general, or even the value of psychotropics (good or bad) in a specific patient. In the more subtle cases of cosmetic psychopharmacology a lack of ethical convergence is reinforced by a relative absence of empirical data. Nevertheless, in particular instances we can reach reasonable decisions - some lives are better than others. Relevant considerations for moral reasoning-imagining about enhancements include both the potential value of stoic acceptance of life's fate (including imperfections and aging, day-to-day distress, and idiosyncracies in personality), as well as the potential value of attempts to change the hand one is dealt (whether by virtue of pharmacotherapy, psychotherapy, or non-medical means). Decisions about whether or not to use psychotropics are likely to remain controversial and given the relative complexity and difficulty of such decisions, this is appropriate.

### Concluding notes on the method

In trying to formulate a conceptual framework, rather than attempting to resolve well-known philosophical debates in their own terms, I have asked whether an integrative approach that uses some constructs drawn from various proponents, can be supported by data from contemporary cognitive-affective science. Although this would not be a widely accepted approach, many have argued that philosophy must increasingly address scientific data. An approach which emphasizes the value of scientific data has perhaps won the battle for questions about the ultimate nature of matter, where philosophy in the absence of physics is no longer acceptable [30]. Similarly, there is growing interest in empirical epistemology (or neurophilosophy) [31,32], and the integration of science with ethics (or neuroethics) [33,34].

Any attempt to include a whole range of thinkers under a simple rubric (such as "classical" or "critical") must fail, and the aim of the contrasts drawn here is to attempt to show some family resemblances, rather than to stake out necessary and sufficient criteria for belonging to divergent schools of thought. Indeed, prototypes and metaphors may be useful not only in day-to-day life, but also lie at the heart of many of our most sophisticated scientific and philosophical constructs. Thus, good scientists use a mixture of conceptual thinking, empirical data and scientific values to make judgments about when to shift paradigms or change classifications. Analogously, Johnson [17] has argued that the concept of morality is itself a radial category. Again, good clinicians use a mixture of conceptual thinking, empirical data and scientific values to make judgments about when to use the medical model and offer treatment.

Indeed, the categories of "classical" and "critical" may themselves reflect everyday mappings of crucial differences between notions of objective-disease/explanatory-cause/medical-treatment versus subjective-illness/understanding-reason/moral-acceptance - between choosing a MEDICAL and a MORAL metaphor. It is comforting, perhaps, that there is a certain consistency to the approach here; the strategy of relying on the relevant data from cognitive-affective science is compatible with a view that is sceptical of formal definitions of constructs, and also with the idea of using philosophical constructs as bootstraps to help conceptualize data and to reach valid explanations of the world.

Psychiatric disorders comprise a large portion of the global burden of disease, and there are ongoing efforts to find effective and cost-efficient treatments. Nevertheless, psychiatry has been accused of being either brainless or mindless [35], with conceptual underpinnings that are often simplistic or dualist [36]. This paper tries to work towards providing a more sophisticated account that integrates a range of perennial conceptual tensions in the field (objectivity/subjectivity, explanation/understanding, absolutism/relativism), and so to assist the important goal of putting psychiatry and psychopharmacology on firmer footing.

## Competing interests

DJS has received research grants and/or consultancy honoraria from Abbott, Astrazeneca, Eli-Lilly, GlaxoSmithKline, Jazz Pharmaceuticals, Johnson & Johnson, Lundbeck, Orion, Pfizer, Pharmacia, Roche, Servier, Solvay, Sumitomo, Takeda, Tikvah, and Wyeth.

## About the author

DJS is Professor and Chair, Dept of Psychiatry, University of Cape Town and Professor Extraordinaire, Dept of Psychiatry, University of Stellenbosch.

## Authors' contributions

DJS conceived of and wrote this manuscript.
